# Development and validation of a blocking ELISA for measurement of rabies virus neutralizing antibody

**DOI:** 10.1128/jcm.02049-24

**Published:** 2025-05-14

**Authors:** Yuewen Xiao, Meng Wu, Weidi Xu, Sheng Sun, Tingfang Liu, Yan Liu, Hongwei Li, Na Feng, Changchun Tu, Ye Feng

**Affiliations:** 1 College of Animal Medicine, Jilin University 12510, Changchun, Jilin Province, China; 2 Institute of Bioengineering, Chongqing Academy of Animal Sciences 580592, Chongqing, China; 3 Changchun Veterinary Research Institute, Chinese Academy of Agricultural Sciences, State Key Laboratory of Pathogen and Biosecurity, Key Laboratory of Jilin Province for Zoonosis Prevention and Control 595703 , Changchun, Jilin Province, China; 4 School of Laboratory Medicine and Biotechnology, Southern Medical University 657716 , Guangzhou, Guangdong, China; 5 Jiangsu Co-innovation Center for Prevention and Control of Important Animal Infectious Diseases and Zoonosis, Yangzhou University 38043, Yangzhou, Jiangsu Province, China; 6 State Key Laboratory of Pathogen and Biosecurity, Academy of Military Medical Sciences 602528, Beijing, China; University of California, Davis, Davis, California, USA

**Keywords:** rabies virus glycoprotein, monoclonal antibody, blocking ELISA, neutralizing antibody detection, validation

## Abstract

Enzyme-linked immunosorbent assay (ELISA) is an effective approach for monitoring herd immunity of animals against rabies but not recommended to detect neutralizing antibody response of individual animals in the framework of international travel since its insufficient agreement with the globally recognized rabies virus (RABV) neutralizing tests. In this study, a blocking ELISA to specifically detect anti-RABV neutralizing antibody was developed using the purified RABV glycoprotein (G) expressed in HEK293T cells as coating antigen, and a labeled anti-G neutralizing monoclonal antibody as the blocking antibody. The overall agreement between the blocking ELISA and fluorescent antibody virus neutralization test in detection of clinical serum samples (dogs = 658; cats = 508) was 97.43% (1,136/1,166), with a diagnostic specificity of 95.63% (219/229) and a diagnostic sensitivity of 97.87% (917/937). Further comparison with the commercial ELISA kits and inter-laboratory validation showed that the blocking ELISA had excellent specificity, sensitivity, and reproducibility. In conclusion, the developed method is a potential tool as an alternative to the virus neutralization test for the detection of canine and feline rabies neutralization antibodies following vaccination.

**IMPORTANCE:**

This study establishes a blocking enzyme-linked immunosorbent assay (ELISA) for detecting rabies neutralizing antibodies in dogs and cats, demonstrating high sensitivity, specificity, and no cross-reactivity. This method provides a reliable alternative to conventional neutralization assays, facilitating efficient large-scale rabies vaccination assessment, and thereby strengthening global rabies control efforts.

## INTRODUCTION

Rabies is a fatal zoonotic disease caused by rabies virus (RABV), which infects mammals and causes severe neurological damage (1). Over 99% of human rabies cases globally are caused by dog transmission (2), and the most effective method to control canine rabies is mass dog vaccination to achieve over 70% vaccination coverage of the dog population (3, 4). The effectiveness of mass vaccination in dogs, cats, or other domestic animals can be monitored using serological tests for rabies, which primarily involve rabies antibody testing (5). The World Organisation for Animal Health (WOAH) and World Health Organization (WHO) suggest that rabies virus neutralizing antibody (RVNA) titer ≥0.5 IU/mL is sufficient to prevent infection with RABV (6, 7).

Quantitative assessment of rabies antibodies is typically conducted using virus neutralization tests (VNTs) (8), including the fluorescent antibody virus neutralization test (FAVN) (9) and rapid fluorescent focus inhibition test (RFFIT) (10). However, these methods are complex, time-consuming, and require cell culture with infectious virus handling, making them unsuitable for large-scale seroprevalence surveys. Both WHO and WOAH recognize enzyme-linked immunosorbent (ELISA) as acceptable tests for monitoring the antibody response of vaccinated animals in the framework of rabies control. In Europe, ELISAs are useful tools for monitoring rabies vaccination campaigns in wildlife animals. In China, ELISAs are routinely used at various levels to assess the immune response in dogs post-vaccination, as mandated by the National Animal Disease Surveillance Program. These international and domestic applications highlight the potential of ELISA to complement or even replace classical VNTs (6, 7, 11).

Various ELISA methods for detecting RABV antibodies in animal or human serum have been established in recent years, including indirect, blocking, and competitive ELISA. The indirect ELISA detects antibodies by using a secondary antibody conjugated to an enzyme, while the blocking ELISA measures the ability of test antibodies to block the binding of a known labeled antibody to the target antigen. The competitive ELISA quantifies antibodies by their competition with a labeled reference antibody for binding to the antigen, with signal reduction indicating higher antibody concentration. In previous studies, indirect ELISA based on inactivated and purified RABV G protein and protein A-peroxidase conjugate for dog and cat serum (12), indirect ELISA based on glycoprotein and goat anti-human IgG antibody for human serum (13), competitive ELISA based on a whole RABV vaccine and anti-G protein monoclonal antibody (mAb) for human and dog serum (14), and blocking ELISA based on unpurified G protein prepared from inactivated RABVs and biotinylated anti-rabies antibodies for domestic and wild carnivores (15, 16). These methods detect IgG and cannot specifically and accurately measure neutralizing antibodies, resulting in a low agreement with VNT results (14, 16, 17). Some methods have adjusted the cut-off values to improve the agreement, such as Zhao et al. (14) who changed the cut-off from 0.5 IU/mL to 1.5 IU/mL. Although this increases specificity, the comparison with VNT results was inaccurate.

Neutralizing antibodies are the only measurable standard to ensure the effectiveness of vaccine-induced immunity (18) and the mandatory passport for international travel of dogs and cats (7). RVNA responses are primarily directed at the virus glycoprotein, which has multiple neutralizing antigenic epitopes, enabling the G protein alone to induce sufficient neutralizing antibodies upon immunization (18). Therefore, a blocking or competitive ELISA for measuring neutralizing antibodies can be established using the G protein and highly potent G protein-specific neutralizing mAbs.

In recent years, the blocking or competitive ELISA methods have been developed using inactivated rabies virus (19) or purified rabies virus-like particles (20) as antigens, and anti-G mAbs as antibodies. Both methods were validated using the mouse neutralization test (21) but not showing agreement. Here, a blocking ELISA for detection of RVNA has been established by using recombinant G protein and a highly potent anti-G neutralizing mAb. The assay was validated by testing a large number of FAVN-tested sera and by an interlaboratory test to determine reproducibility.

## MATERIALS AND METHODS

### Protein, blocking antibody, and serum samples

The purified RABV recombinant glycoprotein (RABV-G) of strain CTN-1V5G (Genbank number: JN234418) expressed in HEK293T cells by the lentiviral vector was prepared at a concentration of 0.064 mg/mL, according to a previous publication (22). The neutralizing antibody used for blocking in this assay is a fully human IgG mAb 25-6C (0.11 mg/mL), which was recently generated through immunizing humanized CAMouse^HG^ mice (23). This mAb was RABV-G protein specific and has broad spectrum reactivity and robust neutralizing activity against eight RABV strains in five subclades (24). The mAb 25-6C was conjugated with horseradish peroxidase (HRP) using an HRP conjugation kit (D601047, Sangon Biotech, China) according to the manufacturer’s instructions. The conjugated mAb 25-6C (HRP-25-6C) is then dialyzed against PBS and stored in 50% glycerol at −20°C for use after determination of its concentration. The WOAH reference serum of dog origin was purchased from Nancy Laboratory for Rabies and Wildlife, France (25). One reference RABV positive control canine serum (PC) with RVNA titer 5 IU/mL, three repeat control sera with RVNA titer 4.5, 1.5, and 0.5 IU/mL, and one negative control canine serum (NC, naïve serum) were archived in our laboratory(26) . Canine distemper virus (CDV) and canine parvovirus (CPV) positive sera were generously provided by Dr. Feng Na at Changchun Veterinary Research Institute. A total of 1,356 canine and feline serum samples were collected across China and archived in our laboratory with RVAN titers < 0.5 IU/mL (*n* = 298 including 137 naïve sera) and ≥0.5 IU/mL (*n* = 1058). Of them, 190 were specifically selected as panel one to determine the cut-off value of the method, including <0.5 IU/mL (*n* = 69 including 32 naïve sera) and ≥0.5 IU/mL (*n* = 121), while the remaining 1,166 were used as panel two for the validation, including <0.5 IU/mL (*n* = 229 including 105 naïve sera) and ≥0.5 IU/mL (*n* = 937). All serum samples used in this study were pre-tested to determine neutralizing antibody titers using the FAVN.

### Determination of EC_50_ of the mAb by indirect ELISA

An indirect ELISA was used to determine the median effective concentration (EC_50_) (27) of mAb 25-6C. Briefly, 96-well microplates (42592, Corning Costar, USA) were coated overnight at 4°C with 50 µL/well of HEK293T cell-expressed RABV-G at 2 µg/mL. The plates were blocked with 200 µL/well of 5% bovine serum albumin (BSA) for 1 h at room temperature after washes with phosphate-buffered saline containing 0.05% Tween 20 (PBST). Serial eightfold diluted mAb ranging from 1:16 to 1:2^34^ was added to the wells (50 µL/well) following further washes with PBST and incubated at 37°C for 1 h. After the incubation, the horseradish peroxidase-conjugated mouse anti-human antibody (ab99759, Abcam, UK) was added to each well at the dilution of 1:1,000 in 5% BSA PBS (50 µL/well) and then incubated at 37°C for 1 h. After the final washes, 50 µL/well of 3,3′,5,5′-tetramethylbenzidine (TMB) substrate (Thermo Fisher Scientific, USA) was added for a chromogenic reaction that lasted 7 min in the dark at room temperature. The reaction was halted by the addition of 50 µL/well of 2M H_2_SO_4_, and the absorbance at 450 nm (OD_450_) was measured using a multifunctional microplate reader Infinite M200 (TECAN, Switzerland).

### Establishment of the blocking ELISA

**Fig 1:**
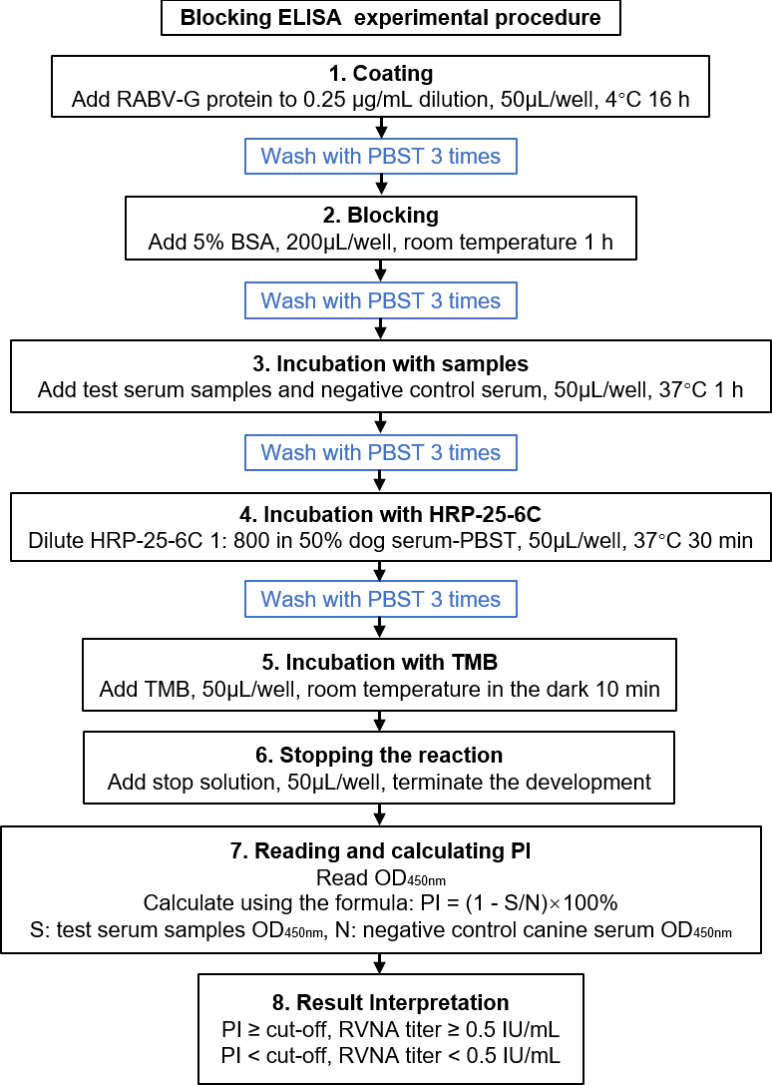
The experimental procedure for blocking ELISA.

The binding specificity of the mAb was determined by Western blotting. In brief, HEK293T cells expressed RABV-G purified by Ni-column affinity chromatography were denatured with SDS loading buffer, resolved by 12% SDS-PAGE alongside a negative control of lysate from normally cultured HEK293T cells, and transferred to a nitrocellulose membrane. After blocking with 5% nonfat milk in PBS for 1 h, the membrane was incubated with mAb 25-6C for 4 h, followed by DyLight 680 Rabbit anti-Human IgG (H + L) secondary antibody (Invitrogen, USA) for 1 h. Detection was performed using an Odyssey imaging system. The optimal concentrations of RABV-G and HRP-25-6C were determined by checkerboard titration blocking ELISA. The RABV-G at 0.25 and 0.5 µg/mL in coating buffer were added respectively to 96-well microplates at 50 µL/well, and then the plates were coated at 4°C overnight, followed by three times PBST washes and blocking with 5% BSA for 1 h at room temperature. After another three washes, 50 µL/well of RABV PC serum (5 IU/mL) and NC serum were separately added to the well, then incubated for 1 h at 37°C. After three times washing, 50 µL/well of twofold serially diluted HRP-25-6C in the range of 1:200–1:800 was added to the plate and incubated for 1 h at 37°C. After three washes, 50 µL/well of TMB was added to the plate for a 10 min chromogenic reaction at room temperature in the dark. After stopping the reaction by adding 50 µL/well of 2M H_2_SO_4_, the OD450 of each well was determined using Infinite M200. The optimal RABV-G coating and HRP-25-6C working concentrations were determined according to the highest percent inhibition (PI) calculated by the formula: PI = (1-OD_450_ value of PC/ OD_450_ value of NC) × 100%. The experimental procedure for blocking ELISA is shown in Fig. 1.

### Determination of cut-off value

The cut-off value of the RABV-G-based blocking ELISA was determined by receiver operating characteristic (ROC) analysis (28) that used 190 canine and cat sera with known FAVN titers respectively being 0.5–13.5 (*n* = 121) and 0.02–0.29 IU/mL (*n* = 69) to maximize both specificity and sensitivity of the method. The PI value of each serum was analyzed by the ROC curve using GraphPad Prism software 8.0 version to present the area under the curve (AUC) at a 95% CI. The cut-off value of each serum corresponding sensitivity and specificity values was used to calculate the Youden index according to the formula: Youden index = sensitivity + specificity − 1. The cut-off value corresponding to the maximum Youden index is the optimal cut-off point. The calculation of the Youden index is one of the most accurate methods to determine the optimal cut-off point for diagnostic methods (29).

### Estimation of the analytic sensitivity and specificity

PC serum (5 IU/mL) of twofold serial dilutions between 1:2–1:256 was used to evaluate the analytical sensitivity of the method. The highest dilution of the reference PC serum producing a PI value greater than the cut-off point was defined as the analytic sensitivity of the blocking ELISA. Meanwhile, CDV and CPV positive sera were used to estimate the analytical specificity of the blocking ELISA.

### Estimation of the repeatability and reproducibility

The repeatability of the blocking ELISA was analyzed by using three repeat control sera (0.5, 1.5, and 4.5 IU/ mL) in triplicate wells in one run. While reproducibility of the method was estimated by three runs in different times. The coefficient of variation (CV) was used to quantify the degree of variation of the blocking ELISA using the following formula: CV = standard deviation (SD) / mean PI value of each positive serum.

### Validation of the blocking ELISA

To validate the blocking ELISA, 658 dog and 508 cat clinical serum samples (1,166 in total) previously tested by FAVN in our laboratory were used, and the agreement (Kappa value) between the two methods was analyzed statistically by SPSS software version 19.0. In addition, two panels of dog and cat sera (25 in each) were selected, with each consisting of five groups based on antibody titers ≤ 0.06 IU/mL (including naïve sera), 0.07–0.49, 0.5–2, 2.01–5, and＞5 IU/mL (five per group). These serum samples were sent as blinded samples to five specialized laboratories in China for the inter-laboratory validation, together with the prepared kits of the methods including testing procedure. The results from the participating laboratories were returned to our laboratory for statistical analysis.

### Comparison of the blocking ELISA with commercial ELISA kits

Two panels of dog and cat sera (100 in each) were selected, with each consisting of five groups based on antibody titers: ≤0.06, 0.07–0.49, 0.5–1, 1.01–5, and 5 IU/mL (20 per group). Details of the kits were included in the Supplementary Table. The experiments using commercial kits were operated based on their instructions respectively. All kits are operated in strict accordance with the instructions.

## RESULTS

### Establishment of the blocking ELISA

**Fig 2:**
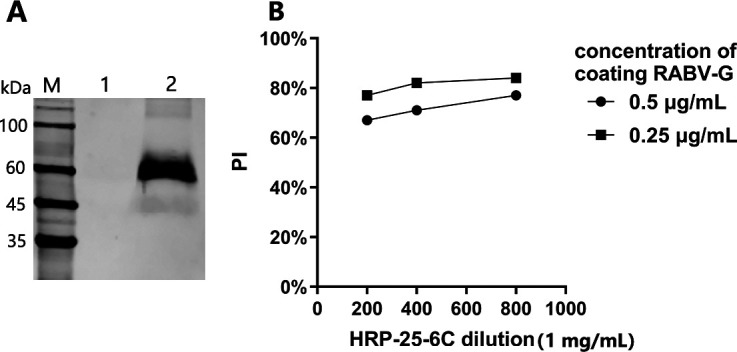
(**A**) Western blotting analysis of glycoprotein with mAb 25-6C. M: Marker, Lane 1: negative control, Lane 2: purified RABV-G lysate. (**B**) The optimal concentrations of antigen RABV-G and HRP-25–6C. The *x*-axis represents the dilution of HRP-25=6C, and the initial concentration of HRP-25-6C is 1 mg/mL; the *y*-axis represents PI; the circular legend represents the RABV-G coated concentration of 0.5 µg/mL; the square legend represents the RABV-G coated concentration of 0.25 µg/mL.

The mAb 25-6C has a broad reactivity and robust neutralizing activity against different RABV subclades and high affinity (1.98 nM) (24). Here, the result of the antigen-binding assay showed this mAb had low EC_50_ (51.78 ng/mL), confirming its high potency. Western blotting in Fig. 2A further showed strong and specific binding of mAb 25-6C to purified RABV-G protein. All these results showed that mAb 25-6C has a strong capacity for establishing the blocking ELISA, in which the combination of 0.25 µg/mL of RABV-G and 800-fold diluted HRP-25-6C (1.25 µg/mL) produced the maximum PI value via checkerboard titration blocking ELISA (Fig. 2B), therefore were selected as working concentrations of antigen and antibody of the method.

**Fig 3:**
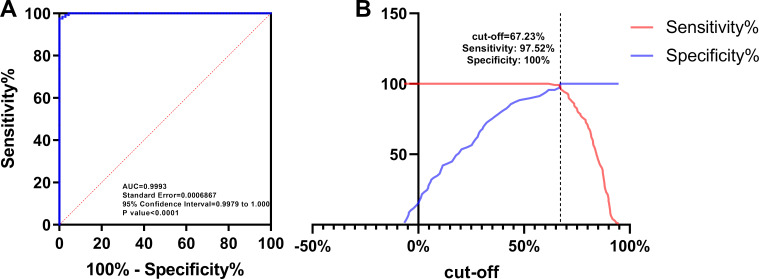
ROC analysis (**A**) The ROC curve was performed to determine the cut-off value of the developed blocking ELISA. (**B**) Different cut-off values correspond to specificity and sensitivity of the blocking ELISA, which were tested by ROC curve analysis.

To determine the cut-off value, two panels of canine and cat sera with known FAVN titers respectively being 0.5–13.5 (*n* = 121) and 0.02–0.29 IU/mL (*n* = 69) were used to maximize the specificity and sensitivity of the method in ROC analysis. As a result, the area under the ROC curve (AUC) for the blocking ELISA was 0.9993 (*P* < 0.0001) with a 95% confidence interval (CI) of 0.9979 to 1.000, showing that the method could effectively differentiate FAVN titers of the sera between <and ≥ 0.5 IU/mL (Fig. 3A) and the PI cut-off value corresponding to ROC curve analysis was 67.23% with 100.00% specificity (69/69) and 97.52% sensitivity (118/121) at the maximum Youden index (Fig. 3B).

### Analytic sensitivity and specificity evaluation of the blocking ELISA

**Fig 4:**
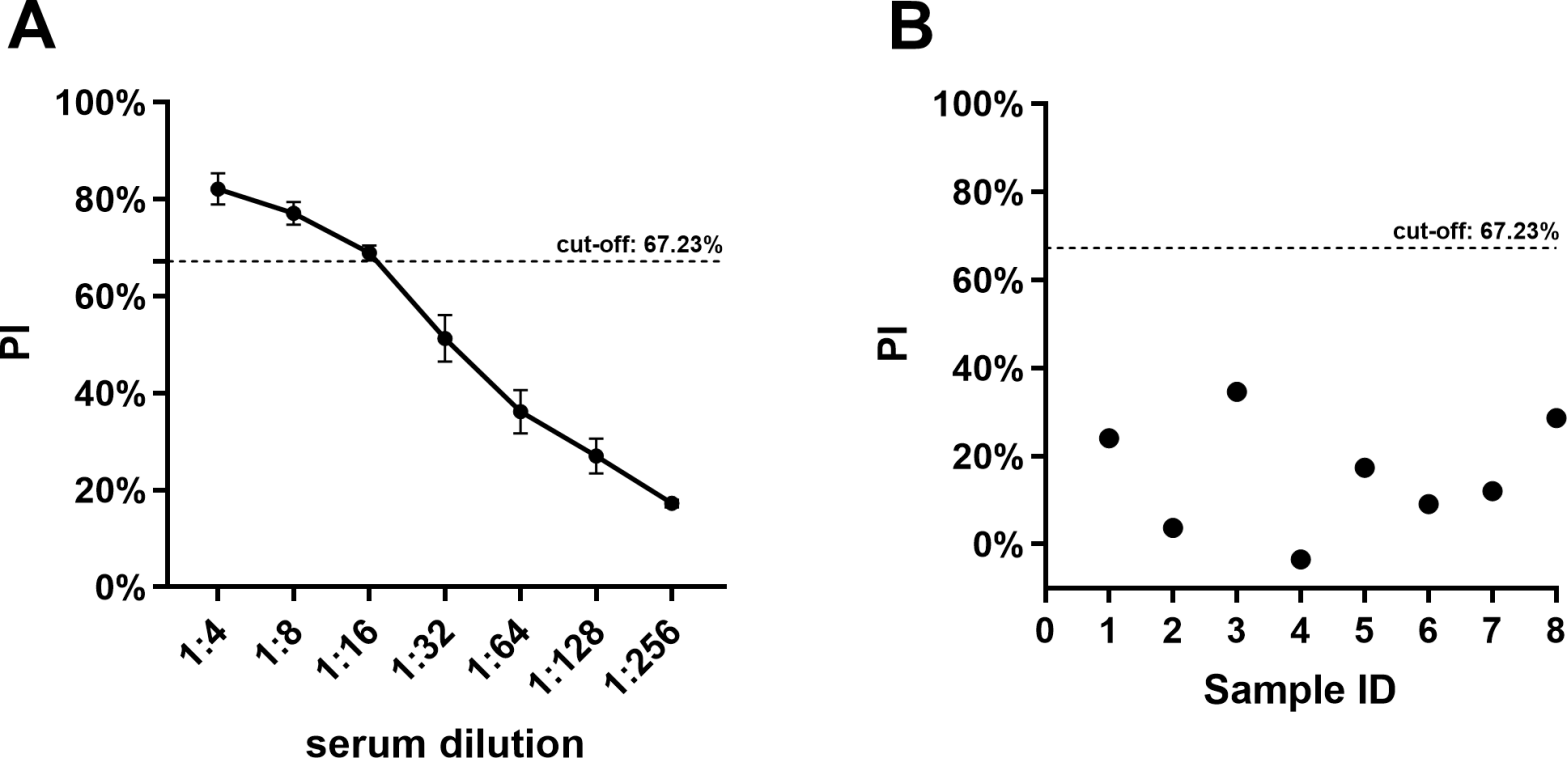
(**A**) Analytic sensitivity of the developed RABV-G based blocking ELISA. Twofold serially diluted PC serum ranging from 1:4 to 1:256 were detected via the ELISA. The PI cut-off value of 67.23% was marked with a dashed line. (**B**) Analytic specificity of the RABV-G based blocking ELISA. Sample ID 1–8 represents 8 CDV and CPV simultaneously immunized dog sera.

To estimate the analytic sensitivity, twofold serial dilutions of PC (5 IU/mL) from 1:4 to 1:256 were detected using the blocking ELISA, and the result showed that the 1:16 dilution was the highest PC dilution to produce the positive result (Fig. 4A). To ensure the specificity of our ELISA method, we tested it against antibodies from commonly administered canine vaccines, such as CDV and CPV, to verify that it does not produce false-positive results through cross-reactivity with non-target antibodies frequently present in clinical samples. The result showed that eight CDV and CPV simultaneously immunized dog sera were tested by the blocking ELISA, and the result showed that their PIs were −3.41%–34.61% (Fig. 4B), much lesser than the cut-off value 67.23%, which demonstrated that the developed blocking ELISA has excellent specificity.

### Repeatability and reproducibility of the blocking ELISA

In the repeatability analysis, three repeat control sera (4.5, 1.5, and 0.5 IU/mL) were used in the blocking ELISA in one plate one run with triplicate. In the reproducibility analysis, three identical repeat control sera were tested in three different plates and three independent runs. As shown in Table 1, the repeatability CV ranged from 0.24% to 2.25%, while the reproducibility CV ranged from 1.24% to 5.22%, indicating excellent repeatability and reproducibility of the blocking ELISA.

**TABLE 1:** Repeatability and reproducibility analyses of the blocking ELISA^*a*^

Control positive serum titer (IU/mL)	Repeatability			Reproducibility		
	Mean	SD	CV	Mean	SD	CV
0.5	68.63%	0.015	2.25%	67.53%	0.035	5.22%
1.5	90.96%	0.002	0.24%	91.24%	0.006	0.61%
4.5	90.66%	0.018	1.93%	91.94%	0.011	1.24%

^*a*^ Mean: the average of PI values from three repeated tests; CV: coefficient variation; SD: standard deviation.

### Comparison of RVNA titers measured by developed blocking ELISA and FAVN

**Fig 5:**
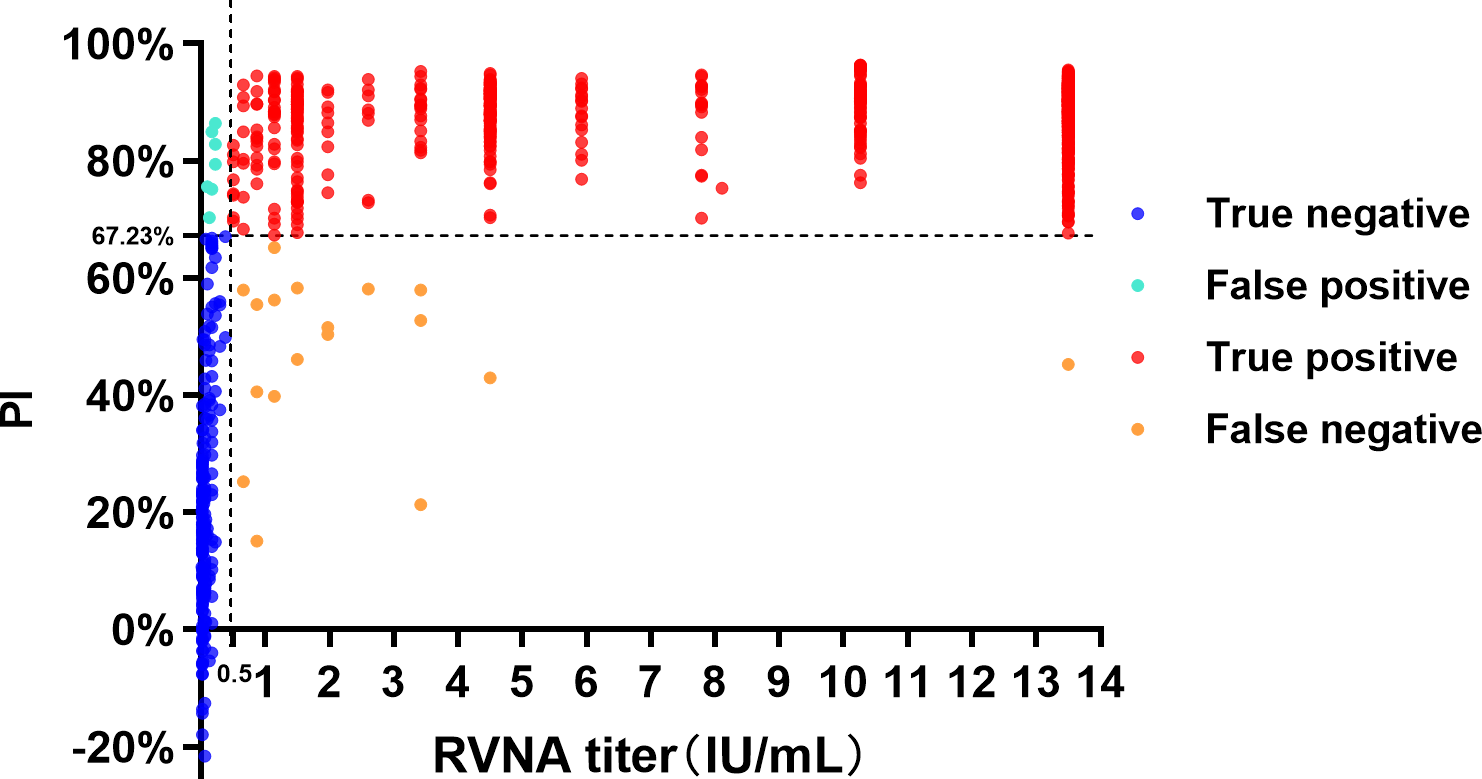
Relationship between RVNA titers and corresponding PI in 1,166 dog and cat serum samples.

To evaluate the diagnostic specificity and sensitivity of the blocking ELISA, 658 canine and 508 cat clinical serum samples previously tested by FAVN (panel two in section 2.1) were used, and the result showed that the concordance, sensitivity, and specificity of canine sera were 96.35%, 96.9% (CI, 95.02%–98.08%) and 94.37% (CI, 89.28%–97.12%), respectively, while those of the cat sera were 98.82%, 99.05% (CI, 97.58%–99.63%), and 97.7% (CI, 92%–99.59%), respectively (Table 2). The Kappa values of canine and feline sera are 0.894 and 0.959 (*P* < 0.001), which indicated a high level of concordance in the detection results of the two methods. The total concordance, sensitivity, and specificity of the blocking ELISA in detection of 1,166 clinical serum samples in comparison with FAVN were 97.43%, 95.63% (CI, 91.88%–97.77%), and 97.87% (CI, 96.66%–98.66%). We have conducted a comprehensive analysis of the relationship between RVNA titers and PI values for 1,166 dog and cat sera in Fig. 5. The overall diagnostic specificity and sensitivity of the blocking ELISA were 95.63% (219/229) and 97.87% (917/937), respectively, as previously shown in Table 2. Spearman correlation analysis revealed a significant positive correlation between RVNA titers and PI values (r = 0.6023, 95% CI: 0.5633–0.6387), indicating a moderate positive relationship with statistically significant relevance (*P* < 0.0001). As our blocking ELISA is a qualitative assay, the PI values reach a plateau when RVNA titers exceed a certain threshold and do not increase further with higher RVNA titers.

**TABLE 2:** Diagnostic specificity and sensitivity of the blocking ELISA obtained from 658 dog and 508 cat sera^*a*^

Sera	ELISA results	FAVN results	
		<0.50 IU/mL	≥0.50 IU/mL
Dog	Specificity (%)	94.37% (134/142)	–
	Sensitivity (%)	–	96.90% (500/516)
	95% CI	89.28%–97.12%	95.02%–98.08%
Cat	Specificity (%)	97.70% (85/87)	–
	Sensitivity (%)	–	99.05% (417/421)
	95% CI	92%–99.59%	97.58%–99.63%
Total	Specificity (%)	95.63% (219/229)	-
	Sensitivity (%)	-	97.87% (917/937)
	95% CI	91.88%–97.77%	96.66%–98.66%

^*a*^ 95% CI: 95% confidence interval. (-): indicates not applicable.

The 1,356 serum samples, obtained from animals immunized with 13 commercial vaccine brands representing 10 distinct vaccine strains, include 1,167 samples with known information, as shown in Table 3. The serum samples were categorized according to the vaccine strains used for immunization. The sensitivity, specificity, and concordance rates of the blocking ELISA were calculated separately for each vaccine strain, and the results are shown in Table 4. For sera from animals immunized with the G52 strain, the sensitivity, specificity, and concordance rates were 97.36% (37/38), 97.83% (225/230), and 97.76% (262/268), respectively. For sera from animals immunized with the SAD strain, the sensitivity, specificity, and concordance rates were 91.89% (34/37), 98.58% (346/351), and 97.94% (380/388), respectively. For sera from animals immunized with the Pasteur RIV strain, the sensitivity, specificity, and concordance rates were 90.32% (28/31), 97.01% (422/435), and 96.57% (450/466), respectively. For the remaining vaccine strains, the number of serum samples was relatively small, but the concordance rates were all 100%.

**TABLE 3:** Vaccine information of 1,167 immune sera

Brand	Manufacturer	Vaccine strain	Animal species	Number of samples
Rabvac 3	Pfizer Zoetis Inc (USA)	SAD	Dogs, cats, horses	317
Rabies vaccine, inactivated	Eli Lilly and Company (USA)	SAD	Animal	66
NOBIVAC RABIES	Merck & Co., Inc. Intervet International B.V. (USA)	Pasteur RIV	Dogs and cats	466
RABISIN	Boehringer Ingelheim Vetmedica GmbH (Germany)	G52	Dogs and cats	91
RABISIN	Merial INC (France)	G52	Dogs and cats	177
Rabies vaccine, inactivated	VIRBAS LABORATORIES (France)	VP12	Animal	11
Rabies (tissue culture) vaccine, inactivated	Nisseiken Co., Ltd. (Japan)	RC·HL	Dogs and cats	2
Quankang	Liaoning Yikang Biological Corporation Limited (China)	Flury	Dogs	10
Ke Youwang	Wuhan Keqian Biology Co., Ltd (China)	SAD	Dogs	4
Rabies vaccine, inactivated	Changzhou Tongtai Biological Pharmaceutical Technology Crop., Ltd (China)	SAD	Dogs	1
FullGuard	Heyuan Bioengineering (China)	CVS-11	Dogs	9
Chong Yijia	Qingdao YEBIO (China)	r3G	Dogs	8
Youkuangbao	UBEN (China)	PV/BHK-21	Dogs	5

**TABLE 4:** Diagnostic specificity, sensitivity, and concordance of the blocking ELISA in detection of 1,167 immunized serum samples

Vaccine strain	Specificity	Sensitivity	Concordance
CVS-11	100.00% (2/2)	100.00% (7/7)	100.00% (9/9)
Flury	100.00% (5/5)	100.00% (5/5)	100.00% (10/10)
G52	97.36% (37/38)	97.83% (225/230)	97.76% (262/268)
SAD	91.89% (34/37)	98.58% (346/351)	97.94% (380/388)
Pasteur RIV	90.32% (28/31)	97.01% (422/435)	96.57% (450/466)
PV/BHK-21	——^*a*^	100.00% (5/5)	100.00% (5/5)
r3G	100.00% (6/6)	100.00% (2/2)	100.00% (8/8)
RC·HL	——	100.00% (2/2)	100.00% (2/2)
VP12	100.00% (4/4)	100.00% (7/7)	100.00% (11/11)

^*a*^ (——): Number of sera consistent with FAVN results/total number of corresponding sera.

### Comparison of the blocking ELISA and commercial ELISA kits

**Fig 6:**
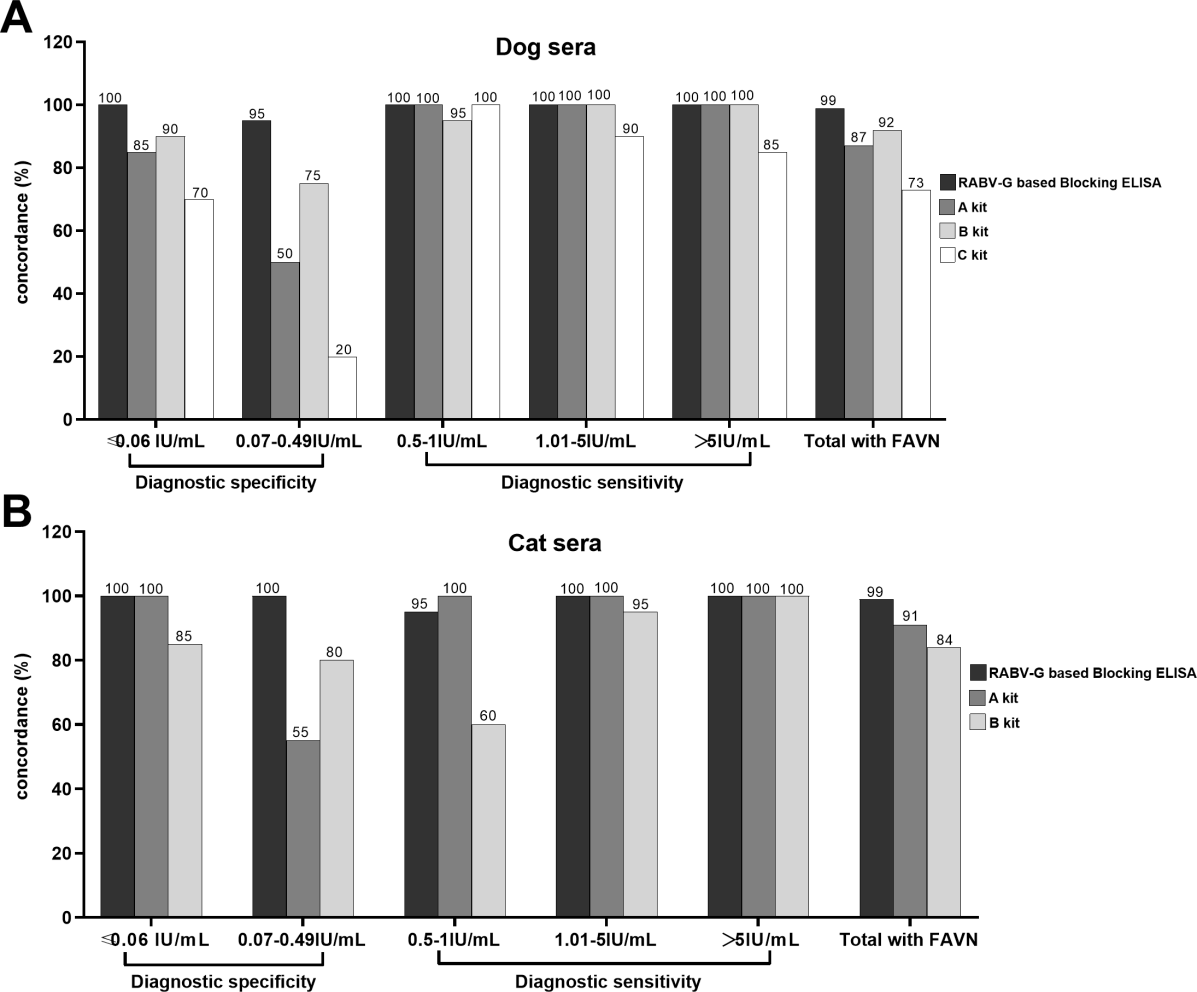
(**A**) Comparison of the developed blocking ELISA and three commercial ELISA kits with FAVN for the detection of RVNA on 100 canine sera. (**B**) Comparison of the developed blocking ELISA and two commercial ELISA kits with FAVN for the detection of RVNA on 100 feline sera. The C kit can only detect canine sera, so it is not tested with cat sera.

The performance of the blocking ELISA was compared with that of three commercial rabies antibody ELISA kits by using 200 selected dog and cat sera (100 for each) with RVNA titers ranging from ≤0.06, 0.07–0.49, 0.5–1, 1.01–5, and ＞5 IU/mL (20 sera in each range, each animal species). As the results showed in Fig. 6, the total concordance between the blocking ELISA and FAVN in detection of dog and cat sera is 99%, significantly higher than that of three commercial kits. The diagnostic specificity of the blocking ELISA as tested by negative sera and the sera of 0.07–0.49 IU/mL was 95%–100%, much higher than that of three commercial kits. The diagnostic sensitivity of the blocking ELISA as tested by the sera of ≥0.5 IU/mL was 95%–100%, it is comparable to Kit A and superior to kits B and C. Kappa consistency analysis was performed for the concordance of each kit with FAVN (Table 5). The blocking ELISA had the highest consistency with FAVN (Kappa = 0.979, *P* < 0.001), followed by kit B (Kappa = 0.748, *P* < 0.001), kit A (Kappa = 0.714, *P* < 0.001), and kit C (Kappa = 0.42, *P* < 0.001). In addition, according to the exact McNemar test, there was no statistically significant difference between the blocking ELISA (*χ^2^* = 0.5, *P* = 0.48), kit B (*χ^2^* = 0.375, *P* = 0.54), and FAVN test results, while kit A (*χ^2^* = 0.045, *P* < 0.0001) and kit C (*χ^2^* = 9.481, *P* = 0.0021) had statistically significant differences with FAVN test results.

**TABLE 5:** Diagnostic specificity, diagnostic sensitivity, coincidence rate, and comparative analysis of blocking ELISA versus three kits in dog and cat sera

Kit	Specificity	Sensitivity	Concordance	Kappa value	χ^2^
Blocking ELISA	98.75% (79/80)	99.17% (119/120)	99% (198/200)	0.979 (*P*＜0.001）	0.500 (*P* = 0.48）
A	72.5% (58/80)	100% (120/120)	89% (178/200)	0.783 (*P*＜0.001）	0.045 (*P*＜0.0001）
B	82.5% (66/80)	91.67% (110/120)	88% (176/200)	0.749 (*P*＜0.001）	0.375 (*P* = 0.54）
C	45% (18/40)	91.67% (55/60)	73% (73/100)	0.395 (*P*＜0.001）	9.481 (*P* = 0.0021）

### Inter-laboratory validation of the blocking ELISA

The result of validating the blocking ELISA by using 25 dog and 25 cat sera in five rabies professional laboratories is in Table 6. As compared with FAVN results, the diagnostic specificity of the blocking ELISA was 95%–100% in four laboratories, while 85% in one laboratory. The diagnostic sensitivity of the blocking ELISA was 100% in four laboratories and 96.67% in one laboratory. The overall concordance in five laboratories was 94%–100%.

**TABLE 6:** Inter-laboratory validation of the blocking ELISA

	Lab A	Lab B	Lab C	Lab D	Lab E
Diagnostic Specificity (%)	95.00% (19/20)	95.00% (19/20)	85.00% (17/20)	100.00% (20/20)	95.00% (19/20)
95% CI	76.39-99.11%	76.39%–99.11%	63.96%–94.76%	83.89%–100%	76.39%–99.11%
Diagnostic Sensitivity (%)	96.67% (29/30)	100.00% (30/30)	100.00% (30/30)	100.00% (30/30)	100.00% (30/30)
95% CI	83.33-99.41%	88.05%–100%	88.05%–100%	88.05%–100%	88.05%–100%
Concordance (%)	96.00% (48/50)	98.00% (49/50)	94.00% (47/50)	100.00% (50/50)	98.00% (49/50)
95% CI	86.54-98.9%	89.5%–99.65%	83.78%–97.94%	92.87%–100%	89.5%–99.65%

## DISCUSSION

Given that over 95% of human rabies cases are caused by rabid dogs all over the world, WHO and WOAH have set the goal to eliminate dog-mediated human rabies globally by 2030 (6). Consequently, the comprehensive annual immunization and vaccination coverage assessments for dogs and cats have become a critical task for animal disease control and prevention departments in the majority of rabies-endemic countries. The current standard VNTs are time-consuming, laborious, and costly, with low throughput; hence, no method has been available as an alternative to VNT. ELISA is a post-immunization surveillance method recommended by WHO and WOAH, and no existing ELISAs have yet been recommended by WOAH for use in dogs and cats (6, 7, 11). This study specifically used purified RABV protective antigen G protein expressed in mammalian cells, in combination with a G protein-specific mAb targeting the neutralizing antigenic site on the G protein (24), and successfully developed the blocking ELISA capable of detecting RVNA. The antigen RABV-G protein used in this study showed excellent immunogenicity as a potential subunit vaccine in the production of strong neutralizing antibodies in immunized experimental pigs (22). The mAb 25-6C specifically targets antigenic site III and exhibited broad-spectrum reactivity with strong neutralizing activity (405–1,215 IU/mg) against eight RABV strains, including standard CVS-11 and seven street viruses of five subclades in three major clades, Cosmopolitan, Arctic-related, and Asia (24). In the study, a large number of archived canine and feline immunized and negative sera of varying antibody titers were used to assess specificity, sensitivity, repeatability, and reproducibility. Validation was also conducted by other specialized laboratories, and comparisons were made with three commercial ELISA kits. The concordance rate of kit A evaluated by Liu (26) and Wasniewski et al. (16) and the concordance rate of kits B and C evaluated by Liu (26) and Gao et al. (30) were lower than that of the blocking ELISA in this study. All results show that the established blocking ELISA showed a better performance than three commercial kits.

The main obstacle for ELISA to detect RVNA is poor specificity, leading to a high false-positive rate based on the 0.5 IU/mL standard. Therefore, the WOAH has not yet recommended any ELISA kit for individual RVNA testing (7). Validation results from 1166 sera with known RVNA titers showed that the developed method achieved 94.37% (134/142) and 97.70% (85/87) specificities, respectively, in dogs and cats (Table 2), which are significantly higher than the tested commercial ELISA kits (Fig. 6). The discordant dog and cat sera are immune sera. Although their RVNA levels are lower than 0.5 IU/mL, they still possess a degree of neutralizing efficacy to provide partial protection to the body (31). The blocking ELISA showed a 100% specificity in the detection of 137 naïve dog and cat sera. Detection near the cut-off value is the primary issue leading to the low concordance between current ELISAs and VNT, with a higher false-positive rate. Our method has significantly overcome this challenge by employing high-quality antigen and highly specific and potent neutralizing mAb, ensuring specificity and sensitivity in the detection of sera near the cut-off value, thereby improving the concordance rate. The concordance rate of 97.43% (1136/1166) observed in canine and feline sera (Table 2) surpasses the rates previously reported by Wasniewski et al., who utilized the Biopro kit—recommended for assessing rabies vaccination in foxes and raccoon dogs—achieving a rate of 90.7% (912/1,005) (15). Additionally, the Platelia II kit showed an even lower concordance at 80.4% (477/593) (32), and Lobanova VA et al. reported a rate of 89.78% (123/137) using a competitive ELISA method (33). This indicates that the current method outperforms these previous assessments in terms of concordance.

In this study, a blocking ELISA was developed to specifically detect anti-RABV neutralizing antibodies using the purified G protein from the RABV vaccine strain CTN-1V5G as the coating antigen. Compared with the G protein of the CVS-11 strain, the CTN-1V5G G protein demonstrated superior binding capability to the monoclonal antibody 25-6C in preliminary experiments (data not shown). The blocking ELISA based on RABV G protein epitope I established with this G protein exhibited excellent performance in detecting 200 clinical dog serum samples, showing a sensitivity of 91.18% and a specificity of 99.15% compared with the FAVN (34). While considering that 0.5 IU/mL is the critical value for determining whether the vaccinated individual has reached effective protection against rabies (6, 7), the RVNA titer slightly lower than 0.5 IU/mL, and higher than that (0.5–1 IU/mL) has a major impact on the specificity and sensitivity of ELISA. Therefore, this study developed an ELISA method based on the rabies virus glycoprotein epitope III. Glycoprotein epitope III is highly conserved among common vaccine strains, such as Flury, CVS-11, RC·HL, PV, and SAD. By leveraging the advantages of the CTN-1V5G G protein to ensure reliable and accurate detection of neutralizing antibodies to evaluate a large number of serum samples with titers between 0 and 1 IU/mL. Therefore, in our assessment of the kits and inter-laboratory validation of the blocking ELISA in this study, sera with antibody titers within the 0–1 IU/mL range are included. Current ELISA kits are unable to achieve high specificity in the range of 0–0.49 IU/mL and high sensitivity in the range of 0.5–1 IU/mL (see Fig. 6), so they cannot replace VNT. However, our method demonstrates a high concordance with VNT, particularly in sera with titer 0.07–0.49 IU/mL; the concordance with FAVN is 95% (19/20 dogs) and 100% (20/20 cats), while kits A, B, and C’s concordance with FAVN is 50%, 75%, and 20% in dogs, and kits A and B’s concordance with FAVN is 55% and 80% consistent with FAVN in cats, respectively (Fig. 6). The blocking ELISA established in this study, characterized by high sensitivity, strong specificity, outstanding concordance with FAVN, and rapid detection. To develop its international use, we will seek validations from professional rabies laboratories, including reference laboratories in other countries, to further confirm its excellent concordance with VNTs.

### Conclusions

A RABV-G based blocking ELISA was developed to detect RVNA. The blocking ELISA exhibited excellent diagnostic sensitivity, diagnostic specificity, and repeatability in the detection of RVNA. Furthermore, it did not cross-react with antisera against CDV and CPV. It also showed excellent coincidence compared with the three kits sold in the market and great diagnostic specificity, sensitivity, and consistency rates in five laboratories. To sum up, the RABV-G based blocking ELISA can offer simplicity, speed, and low cost and has the potential for large-scale monitoring of serum immune responses in dogs and cats after vaccination.

## SUPPLEMENTAL MATERIAL

Tables S1 and S2 jcm.02049-24-s0001.docx Table S1: Information of three commercial antibody ELISA kits (Biopro Rabies ELISA Ab Kit, Czech Republic; Jinnuo Diagnosis Rabies Virus Antibodies ELISA Test Kit, China and Yian-Bio ELISA Kit for Rabies Virus Antibodies, China). Table S2: Information of 1356 serum samples.

ASM does not own the copyrights to Supplemental Material that may be linked to, or accessed through, an article. The authors have granted ASM a non-exclusive, world-wide license to publish the Supplemental Material files. Please contact the corresponding author directly for reuse.
