# Supplementary material for: Development and validation of a blocking ELISA for measurement of rabies virus neutralizing antibody
Source: J Clin Microbiol. 2025 May 14;63(6):e02049-24. doi: 10.1128/jcm.02049-24 (PMC12153311; doi:10.1128/jcm.02049-24)
Supplement: Tables S1 and S2 — Table S1: Information of three commercial antibody ELISA kits (Biopro Rabies ELISA Ab Kit, Czech Republic; Jinnuo Diagnosis Rabies Virus Antibodies ELISA Test Kit, China and Yian-Bio ELISA Kit for Rabies Virus Antibodies, China). Table S2: Information of 1356 serum samples. [file jcm.02049-24-s0001.docx]

Supplementary Table 1. Information of three commercial antibody ELISA kits

| **Kit code** | **Kit name** | **Lot No.** | **Type** | **Targeted animal** | **Antigen** | **Antibody** | **Specificity** | **Sensitivity** | **Concordance** |
| --- | --- | --- | --- | --- | --- | --- | --- | --- | --- |
| **A** | Biopro®, Rabies ELISA Ab Kit (Czech Republic) | R2308 | blocking | domestic and wild carnivores | RABV G protein | biotinylated anti-RABV antibodies | 48.68%  (37/76) ^(26)^  99.49%  (395/397) ^(17)^ | 97.37%  (111/114) ^(26)^  85%  (517/608) ^(17)^ | 77.89%  (148/190) ^(26)^  90.7%  (912/1005) ^(17)^ |
| **B** | Jinnuo Diagnosis, Rabies Virus Antibodies ELISA Test Kit (China) | 20240130 | indirect | domestic and wild carnivores | inactivated RABV | HRP-labeled anti-RABV antibody | 47.37%  (36/76) ^(26)^  29.5%  (48/163) ^(30)^ | 86.84%  (99/114) ^(26)^  96.3%  (289/300) ^(30)^ | 71.05%  (135/190) ^(26)^  72.8%  (404/463) ^(30)^ |
| **C** | Yian-Bio, ELISA Kit for Rabies Virus Antibodies (China) | Y202312011A | indirect | dog | inactivated RABV | HRP-labeled sheep anti-dog IgG antibody | 48.68%  (37/76) ^(26)^  52.8%  (86/163) ^(30)^ | 88.6%  (101/114) ^(26)^  81.3%  (244/300) ^(30)^ | 72.63%  (138/190) ^(26)^  71.3%  (330/463) ^(30)^ |

Supplementary Table 2. Information of 1356 serum samples

| Immunization status | Vaccine strain | RVNA titer group (IU/mL) | Number | Total |
| --- | --- | --- | --- | --- |
| Yes | CVS-11 | 0-0.49 | 2 | 9 |
|  |  | 1.01-5 | 5 |  |
|  |  | ＞5 | 2 |  |
|  | Flury | 0-0.49 | 5 | 10 |
|  |  | 1.01-5 | 4 |  |
|  |  | ＞5 | 1 |  |
|  | G52 | 0-0.49 | 38 | 268 |
|  |  | 0.5-1 | 16 |  |
|  |  | 1.01-5 | 76 |  |
|  |  | ＞5 | 138 |  |
|  | HCP-SAD | 0-0.49 | 35 | 383 |
|  |  | 0.5-1 | 27 |  |
|  |  | 1.01-5 | 95 |  |
|  |  | ＞5 | 226 |  |
|  | SAD | 0-0.49 | 2 | 5 |
|  |  | ＞5 | 3 |  |
|  | Pasteur RIV | 0-0.49 | 31 | 466 |
|  |  | 0.5-1 | 31 |  |
|  |  | 1.01-5 | 109 |  |
|  |  | ＞5 | 295 |  |
|  | PV/BHK-21 | 1.01-5 | 2 | 5 |
|  |  | ＞5 | 3 |  |
|  | r3G | 0-0.49 | 6 | 8 |
|  |  | ＞5 | 2 |  |
|  | RC·HL | ＞5 | 2 | 2 |
|  | VP12 | 0-0.49 | 4 | 11 |
|  |  | 1.01-5 | 4 |  |
|  |  | ＞5 | 3 |  |
|  | Unknown | 0-0.49 | 2 | 7 |
|  |  | 0.5-1 | 2 |  |
|  |  | ＞5 | 3 |  |
| No | —— | 0-0.49 | 137 | 137 |
| Unknown | Unknown | 0-0.49 | 36 | 45 |
|  |  | 0.5-1 | 4 |  |
|  |  | 1.01-5 | 3 |  |
|  |  | ＞5 | 2 |  |
